# Supplementary material for: Factors associated with U.S. adults’ willingness to allow teenagers to play tackle football
Source: PLoS One. 2022 Sep 7;17(9):e0273229. doi: 10.1371/journal.pone.0273229 (PMC9451093; doi:10.1371/journal.pone.0273229)
Supplement: S1 Appendix — (DOC) [file pone.0273229.s001.doc]

**S1 Appendix. Demographic Response Options**

| **Variable** | | **Values** |
| --- | --- | --- |
| Age | | Actual age in years |
| Education (4 categories) | | 1 = Less than HS |
|  |  | 2 = HS |
|  |  | 3 = Some college |
|  |  | 4 = Bachelors degree or higher |
| Gender | 1 = Male | |
|  | 2 = Female | |
| HH Income (profile and imputed) | 1 = Less than $5,000; 2 = $5,000 to $7,499 | |
|  | 3 = $7,500 to $9,999; 4 = "$10,000 to $12,499 | |
|  | 5 = $12,500 to $14,999; 6 = "$15,000 to $19,999 | |
|  | 7 = $20,000 to $24,999; 8 = $25,000 to $29,999 | |
|  | 9 = $30,000 to $34,999; 10 = $35,000 to $39,999 | |
|  | 11 = $40,000 to $49,999; 12 = $50,000 to $59,999 | |
|  | 13 = $60,000 to $74,999; 14 = $75,000 to $84,999 | |
|  | 15 = $85,000 to $99,999; 16 = $100,000 to $124,999 | |
|  | 17 = $125,000 to $149,999; 18 = $150,000 to $174,999 | |
|  | 19 = $175,000 to $199,999 20 = $200,000 to $249,999 | |
|  | 21 = $250,000 or more | |
| Total no. of HH members age 1 or younger | Number of household members in age group | |
| Total no. of HH members age 2 to 5 | Number of household members in age group | |
| Total no. of HH members age 6 to 12 | Number of household members in age group | |
| Total no. of HH members age 13 to 17 | Number of household members in age group | |
| Total no. of HH members age 18 or older | Number of household members in age group | |
