# Supplementary material for: Factors associated with U.S. adults’ willingness to allow teenagers to play tackle football
Source: PLoS One. 2022 Sep 7;17(9):e0273229. doi: 10.1371/journal.pone.0273229 (PMC9451093; doi:10.1371/journal.pone.0273229)
Supplement: S2 Appendix — (DOCX) [file pone.0273229.s002.docx]

**S2 Appendix. All Sport Based Survey Items**

**Q1: When you were in high school, which of the following sports did you play at the varsity level for at least one season (select all that apply)?**

1. Baseball

2. Basketball

3. Cross Country

4. Football

5. Soccer

6. Softball

7. Swimming & Diving

8. Tennis

9. Track & Field

10. Volleyball

**Q2: Knowledge of Football Safety Measures Quiz**

(correct response in parentheses: True/False)

1. In 2016, a Division 1 conference in the U.S. eliminated tackling in all football practices. (True)
2. In 2018, the NCAA reduced the number of preseason practice sessions. (True)
3. In 2019, the National Federation of High School Associations implemented a policy that after a player reaches a maximum number of tackles in a high school football game, the player must sit out the rest of the game. (False)
4. In college football, a player that leads with his head to hit another player can be ejected without warning. (True)
5. There is currently a football helmet available to decrease the number of head impacts players sustain in practices and games. (False)
6. In 2019, Pop Warner eliminated all kickoffs from youth football games. (True)
7. The National Federation of High School Associations passed a policy eliminating all tackle football for middle school/junior high levels starting in 2025. (False)
8. USA Football, the national governing body over amateur football in America, developed 5 levels of contact in 2014 to guide the intensity of football practice. (True)
9. USA Football issued a rule in 2019 eliminating tackling for elementary age youth football leagues. (False)
10. Since 2002, the National Football League has made at least 50 rules changes intended to eliminate potentially dangerous tactics and reduce the risk of injuries. (True)

**Q3: To what extent to do you agree with the following statements?**

1. Concussions in football are a serious problem.
2. As a parent of a teenage boy, or if I was a parent of a teenage boy, I would let him play tackle football if he wanted to.
3. The media has exaggerated the problem of concussions in football.
4. The rule changes implemented for football games in recent years have made football a safer sport.
5. I consider myself a football fan.

Likert scale:

1. Strongly Disagree
2. Disagree
3. Neither Agree nor Disagree
4. Agree
5. Strongly Agree

**Q4: How many players out of 100 do you estimate would get a concussion during 1 season of high school football?**

Please write in your response. (Range 0-100)

**Q5: Indicate how much you worry about each statement for your own teenage boy (or how much you would worry if you had a teenage boy).**

1. My child getting a concussion while playing tackle football in high school.
2. My child having long-term negative effects on the brain from playing tackle football through high school.
3. My child suffering from dehydration or heat-related illness when playing tackle football in high school.
4. My child getting too many head injuries from playing tackle football in high school.
5. My child breaking a bone while playing tackle football in high school.

Likert scale:

1. I don’t worry at all
2. I worry only a little
3. I worry some
4. I worry a lot
