# Supplementary material for: Factors associated with U.S. adults’ willingness to allow teenagers to play tackle football
Source: PLoS One. 2022 Sep 7;17(9):e0273229. doi: 10.1371/journal.pone.0273229 (PMC9451093; doi:10.1371/journal.pone.0273229)
Supplement: S1 Table — (DOCX) [file pone.0273229.s004.docx]

**Supplemental Table 1. Likert Item Distribution**

|  | Strongly Disagree | Disagree | | Neither Agree nor Disagree | | Agree | | Strongly Agree |
| --- | --- | --- | --- | --- | --- | --- | --- | --- |
| **Outcome variable** | | | | | | | | |
| Willingness to allow teenage boys to play football | 136 (14.5) | 139 (14.8) | | 297 (31.7) | | 303 (32.3) | | 63  (6.7) |
| **Belief-based items** | | | | | | | | |
| Belief media exaggerates the concussion problem. | 264 (28.1) | 294 (31.3) | | 308 (32.8) | | 56  (6.0) | | 16  (1.7) |
| Belief recent rule changes have made football safer | 39  (4.2) | 82  (8.7) | | 472 (50.3) | | 313 (33.4) | | 32  (3.4) |
| Belief concussions in football are a serious problem. | 31  (3.3) | 8  (0.9) | | 98  (10.4) | | 358 (38.2) | | 443  (47.2) |
| **Worry about head injury items** | | | | | | | | |
|  | I don’t worry at all | | I worry only a little | | I worry some | | I worry a lot | |
| My child getting a concussion while playing tackle football in high school. | 117 (12.5) | | 147 (15.7) | | 358 (38.2) | | 316 (33.7) | |
| My child having long-term negative effects on the brain from playing tackle football through high school. | 131 (14.0) | | 152 (16.2) | | 302 (32.2) | | 353 (37.6) | |
| My child getting too many head injuries from playing tackle football in high school. | 131 (14.0) | | 142 (15.1) | | 327 (34.9) | | 338 (36.0) | |
| Note: Values presented as frequency (weighted %). | | | | | | | | |
