# Supplementary material for: Factors associated with U.S. adults’ willingness to allow teenagers to play tackle football
Source: PLoS One. 2022 Sep 7;17(9):e0273229. doi: 10.1371/journal.pone.0273229 (PMC9451093; doi:10.1371/journal.pone.0273229)
Supplement: Attachment — Submitted filename: Reviewer Feedback_PLOS ONE_willingness to allow teens to play football.docx [file pone.0273229.s006.docx]

General Feedback:

Thank you for the opportunity to review this manuscript. It was very well written, easy to follow, and is an important contribution to the literature. I have suggested only minor revisions as detailed below.

Specific Feedback:

Introduction –

Very well written; clear and easy to follow. The introduction develops the important narrative necessary for background understanding of the present study.

Methods –

Would the authors please report the number of individuals that were contacted by Ipsos to complete the survey so that the reader can gauge the response rate? If that information is not available, please say so.

The authors should also provide details on the length of the survey (e.g., was it just the questions for this study or were there other items included for other studies?).

Further, would the authors please report whether there were key demographic factors that were associated with missing data on the received surveys? For example, were older individuals less likely to complete the instrument?

Why were the worry-based items graded on a 4-point Likert scale when the belief-based items were rated on 5-point Likert scales?

Would the authors please clarify if the education level of “attended college (62.5%)” refers only to those who completed a degree program, or if it includes those who may have attended some college without obtaining a post-secondary degree?

Why was household income dichotomized at $75k? Was this a median split?

Results –

For transparency’s sake, I would encourage the authors to also report the frequency of each Likert scale response to all questions, either in Table 1 or in supplemental materials. I understand this is asking for some tedious work, but feel it may be useful for yours and others’ future work that may utilize similar questionnaires. I apologize if this has already been done; I was unable to successfully open the link to the appendix in the review pdf file.

I’m assuming the beta values in Table 2 are standardized. Would you please make this clear in the table note and in the main text where “Est = “ is presented?

Discussion –

The authors note that one possible explanation for overestimated concussion risk is that it might be associated with increased media attention. Were the associations between independent variables evaluated? Specifically, does perception of greater media exaggeration associate with lower perceived concussion risk in your sample? On a related note, did the authors consider interaction effects among independent variables (e.g., parentage and worries, for example)?

Thank you for addressing the concern regarding only asking about boys’ participation in football in your note.
