# Supplementary material for: Factors associated with U.S. adults’ willingness to allow teenagers to play tackle football
Source: PLoS One. 2022 Sep 7;17(9):e0273229. doi: 10.1371/journal.pone.0273229 (PMC9451093; doi:10.1371/journal.pone.0273229)
Supplement: Attachment — Submitted filename: Response to reviewer comments.docx [file pone.0273229.s007.docx]

| **Response to Reviewers** | |
| --- | --- |
| **Reviewer Comment** | **Response / Revision** |
| Reviewer #1: Lines 18-21: Consider breaking this into two sentences, with the 2nd sentence starting as "Little is known...". This will be more direct for readers. Line 20: Please specify knowledge and attitudes here (i.e., football safety knowledge and football attitudes) for clarity. | Thank you for these comments. We broke the sentence into two as suggested and specified knowledge and attitudes. |
| Line 24: Normally I do not make formatting edits, but believe PLOS One does publish online ahead of print. So, I am providing them where they stick out to provide a cleaner copy for readers. Specifically here, please remove the line break and paragraph indentation for the abstract. | Great point. Line break and indentation removed. |
| Line 26: Similar comment to above, please consider referring to "knowledge" and "attitudes" here and throughout as "football safety knowledge" or similar for reader understanding. I believe this will help comprehension throughout. | Adjustment made here and where “knowledge” was not described as “knowledge of football safety measures” or used more broadly describing knowledge such as within the KAB model (knowledge-attitude-behavior) paragraph. |
| INTRODUCTION: Overall this section provides sound background, rationale, and importance of this study. However, the flow and paragraph use is somewhat confusing, and may be a matter of field/discipline preferences. Edits are provided below to help provide funneling flow. | All Introduction comments implemented, see detailed items below. Thanks for the thoughtful commentary and suggested adjustments. |
| Lines 40-41: Consider revising to "...CDC reported that only 26% of youth met this recommendation." | Excellent edit for brevity, thank you. |
| Line 42: Consider specifying the year(s) the 46% statistic comes from. Mentioning because we are in a COVID-era and physical activity, specifically sport, has been impacted. | Comment added connecting this 2019 data to the previous sentence (citations included). |
| Lines 47-49: Similar comment; please specify year so it's clear to readers. | Revised: “… as of 2019” added. |
| Line 59: Typo in this sentence at this point; believe changing "have developed" to "have been implemented..." will work. | Great catch, thank you. Adjustment made and it reads clearer. |
| Lines 78-83: Consider inserting a couple sentences specifically addressing the concerns of repetitive head impacts/concussion occurring in youth sports. For example, there was initial work suggesting football participation before 12 years old was associated with worse late-life cognitive and behavioral outcomes (Stamm et al. 2015; Neurology). Further, there are relatively high head impact magnitudes (e.g. linear and rotational acceleration) and head impact rates (e.g. number of head impacts players experience in a typical practice or competition) experienced in youth tackle football. Lastly, speaking to the national legislation rather than 1 state may provide more implications. For example, there are currently 6 states with active/pending legislation for minimizing contact practice times and/or banning tackle football before a certain age (see <https://brainlaw>. com/youth-tackle-football/). I believe this will enhance the rationale for "why" this study is needed. | Excellent points. Initial youth football participation comment added along with commentary highlighting cumulative season-long head impact frequencies and magnitudes. Stamm et al. 2015 citation included.  Regarding the suggested national emphasis rather than a single state, great point and we made the change to address national legislation rather than California-only and added the referenced brainlaw.com citation. |
| Lines 86-89: Consider adjusting the flow of this introduction so that your research question comes in the final paragraph of the introduction. As is, the research question is provided, and then further rationale behind it is provided. It may be easier for reviewers to be funneled from the overarching context, the underlying rationale, and then finally to the specific research question as is common in many fields. | Thanks for this insight. We made the corresponding adjustment by moving the research question down to the final paragraph of the intro and followed it up with hypotheses. |
| Lines105-122: This information is a bit long and unnecessary to the majority of readers, and also may be more appropriate in the methods section (since this is justification for a methodological decision). The authors should consider summarizing it into ~2 sentences stating (paraphrasing) that they used a KAB model which has been commonly employed in numerous health promotion applications to understand how unique social constructs relate to health behaviors. As a reader and reviewer, this is much more direct and gets the same point across. In the limitations, you can also acknowledge this decision was used and the mixed evidence surrounding its general applicability. | Great point, we consolidated this section to two sentences as suggested and moved to the Methods section. This provided a helpful and significant trim. This limitation was not added because other limitations felt more pressing, but we would be happy to add it if the reviewer feels it would be helpful after reading the much revised version. |
| Lines 123-127: Please remove this information as nothing new is added with this text that is not already mentioned in the intro/methods already. | Removed, thanks for decreasing redundancy. |
| Lines 127-130: Consider moving these hypotheses right after your specific aims/research question for improved flow. | Great point, hypotheses moved to right after the research question, which was also moved. |
| METHODS: The methods overall are well-written. The reordering of paragraphs and some further details are warranted. Lastly, greater details and justification for the statistical modeling are warranted due to points raised below. | Excellent comments, thanks for taking the time to do this. We have attempted to resolve each point line-by-line below. |
| Line 133: Please specify this was a "prospective, cross-sectional survey study." This will be useful information for readers, as well as future research systematically reviewing work on this topic. | Thank you for pointing that out, adjustment made. |
| Lines 136-137: Please 1) move this text to the data analysis section to better inform readers this was performed, and 2) make sure the language surrounding population weighting matches the text on lines 236-238. | Moved this text to the data analysis section prior to modeling commentary. Combined with initial text from lines 236-238 to remove redundancy. |
| Lines 156-157: Please also add the median score here for further support of the Gaussian distribution. | Median score of 3 added. |
| Lines 150: Please consider: 1) changing the "measures" here to "Survey Outcomes". 2) provide any supporting evidence for the development, reliability, validity of this survey for addressing this research question. As is, it is (I believe) touched upon within each predictor's paragraph by specifying a Cronbach's alpha and other text, but believe a standalone paragraph with all this information (likely at the end of the survey outcome section) would be more appropriate. 3) state the order in which all the survey items/sections were administrated so it could be replicated in the future. It would be beneficial to have a couple lines of text explicitly stating the order and components of the survey as the "Survey Outcomes" opener, and then provide the survey item paragraphs in the order they were presented to respondents. | Thank you for this improvement. We have updated the heading to match your suggestions and added a reliability/validity paragraph providing further details about the Cronbach’s alpha and item creation for the knowledge items. This paragraph has been added after the individual item sections within the newly created Survey Outcomes section.  Thanks for the thoughtful readability comment number #3. As suggested we added an order-based sentence to the Survey Outcomes opener to enhance reproducibility and added an Appendices with demographic and sport-specific survey items. |
| Lines 154-155: Can the author please provide their rationale for using a 5-point Likert scale here rather than a binary yes/no for this outcome variable? I foresee this being a point of criticism for interpreting the findings and their implications For example, what do the beta-values presented in Table 2 associating with a 1-point Likert change in willing to allow tackle football participation mean for stakeholders? What is considered an important change? 1 or more-Likert point, any change? | Thanks for mentioning this. Rationale added to text about our interest in greater variance (information) in the outcome which helped us interpret a wider range of possible responses and differences in opinion to the proposed question about allowing participation in tackle football. For the second point, interpretation of a 1-point Likert scale change in this context is a limitation and we added this to the limitations section. |
| Line 181: Why was the "worry about head injuries" category the only one examined on a 4-point Likert? | This is a good question. The parental worry questions were a separate set of items, all of which were measured on a four-point Likert scale with these response options: I don’t worry at all, I worry only a little, I worry some, I worry a lot. Other questions were asked as a set of agree/disagree statements that had these response options: Strongly disagree, Disagree, Neither agree nor disagree, Agree, Strongly Agree. In our next study on this topic we will be revisiting the item structure based on this exploratory data. We added a clarifying comment to the end of the “worry about head injuries” methodological section. |
| Line 176: As mentioned, I assume this is for reliability/agreement assessed by the authors for these items, but without specific detail it is unclear. A paragraph targeting the survey reliability/validity would be fruitful. | Great advice. Reliability/validity paragraph added providing further details about the Cronbach’s alpha and item creating for the knowledge items. This paragraph has been added after the individual item sections within the Survey Outcomes section. |
| Line 217: the authors mention "full maximum likelihood here" but also mention R and the lme4 package. Mentioning because lme4 used restricted maximum likelihood by default, and want to confirm accurate reporting. Additionally, this sentence should be moved down towards the end of the paragraph as it is out of place when describing descriptive statistics in the next paragraph. | Thanks for checking. You are right about R and the lme4 package by default and we did use full maximum likelihood. Excellent point, we moved this sentence to the end of the Data Analysis Procedure section. |
| Lines 207-215: Please move this paragraph into the "Data Analysis Procedure" section as it is not relevant to the outcome measures. | Good point. Moved this paragraph to Data Analysis Procedure section. |
| Lines 201-206: Please confirm the dichotomization of these outcomes was through survey respones (i.e. binary response) rather than data processing by the authors. If data processing by the authors, please provide the exact response options for these so this survey could be replicated. The authors should consider modifying the S1 appendix to include the entire survey contents rather than just the predictor/outcome variables for full transparency. | Thanks for this comment. Dichotomization of gender was through survey responses. The other dichomotomization was done though data processing. To allow for replication of this study, the raw demographic questions and response options were added as a new S3 Appendix document.  Thanks for pointing that out. We created a new S2 Appendix with the full sport survey to enhance reproducibility. There were also standard demographic questions developed by Ipsos KnowledgePanel that were not included in the S2 Appendix. |
| Line 233: Please revise "p<0.05" to "alpha=0.05" because your threshold is a set value and the p-value threshold is set by your alpha value. | Adjustment made. |
| RESULTS: Findings are accurately reported in relation to the models ran. However, concerns are raised about the model appropriateness given the negligible variance attributed to the state levels. Further details are provided below. | Thanks again, see line-by-line comments and revisions below. |
| Lines 243-250: The authors report the increased likelihood of Type I error as justification for using the multi-level model. However, the ICC value of 0.0023 is considerably smaller than those reported by the referenced simulation study, whose minimum ICC value used was 0.01 (over 4x larger). Without data supporting this decision to include such a small ICC and variance associated with the state level (0.003), it does not appear multi-level modeling is necessary here. This may also increase your likelihood for Type II error. Please consider and revise if necessary. | We appreciate the reviewer comments. Although our ICC value estimate for the null model is considerably small, it is our understanding that the ICC value can become much larger after level-1 predictors are added to the model as a reduction in the residual variance will increase the random intercept variance (i.e., conditional ICC becomes larger). As a result, design effects can become much higher and the effective sample size will become smaller, leading to an increased Type I error rate. We have added a citation to support the view today that best practice is to not simply ignore the clustering effect even with a considerably small ICC (Huang, 2018). Nonetheless, based on the reviewer’s comments, we have conducted single-level regression analyses and found the results to not differ in any significant way. Comments added to the first paragraph of Model Results. |
| Table 1. Please describe how fractions of a frequency are being derived, specifically for the demographic counts. Is this due to the national weighting applied? Please confirm this level of decimal precision is needed, otherwise, please round to nearest whole number for frequencies to avoid confusion. | Great point, thank you. Yes, the fractions were initially from the sample weighting, but we have adjusted to round to the nearest whole number as that improves the simplicity and understanding of Table 1. |
| Table 1. Though some of the predictors/outcomes appear to be normally distributed, it may be optimal to also report their median and range, or the frequency (%) for each Likert category for full transparency. | Excellent suggestion. We have created and uploaded this information with frequencies and percentages in a new Supplemental Table 1. |
| Table 2. Please remove the "note" section (leave abbreviations) in this table, and instead change the table title to "Willingness to Allow High School Tackle Football Participation Regression Models. | Good suggestion, we made the adjustment. |
| DISCUSSION: The discussion section provides accurate interpretation of the findings observed. However, the noted statistical model concerns have the potential to alter the findings. Thus only broad review is provided here as a 2nd round of review is warranted. | Thank you for these comments. For the ICC and modeling comments we would be happy to provide more information and look closer at those comments. To our understanding even extremely low ICC values are not safe to ignore so we have added data (Huang, 2018) to support this modeling strategy. This article provides detailed discussion about using multilevel modeling with a low ICC. |
| Lines 327-328: The reference indicating 5 concussions per 100 athletes is an outdated (published in 2000) sport epidemiology report. Sport concussion prevalence is considerably higher now, though is not presented as # per athlete as this fails to account for athlete-exposure. Please see publication by Chandran et al. 2021 in the American Journal of Sports Medicine for more current concussion rates. | Excellent point, we’ve updated the text to include the Chandran et al. 2021 estimate of 6.99 per 10,000 athletic exposures. If you think we need to delete the 2000 estimate, we can definitely do that, but it was kept in to give a similar type of comparison to the question which regular adults could potentially relate to more since they would not know how to comprehend 10,000 AE’s on their own. Just let us know though if you think the 2000 estimate should be dropped all together. |
| Line 336: Here and throughout, the directionality of the outcome variable association should be provided (e.g. ".... were significantly associated with [increase? decrease?] levels of football participation willingness." | Thank you for this improvement, by clarifying the directionality, the readability of the main discussion findings improved. We made this suggested edit throughout the key findings interpretations in the Discussion. |
| Lines 359-360: The authors should also consider that the survey used may have been too difficult and thus not an accurate measure of football safety knowledge. As a concussion and head impact biomechanics researcher, I would have only gotten 8/10 items correct. | Thanks for mentioning this. We adjusted the sentence about survey limitations in the limitations section to include a comment about the survey potentially being too difficult. |
| Lines 398-403: Consider either removing or adding this note text to where it is referenced. Superscripted notes are often ignored or missed by readers, and thus should be placed above where applicable. | Removed the note and added the commentary on it to the limitations section. |
|  |  |
| Reviewer #2: General Feedback: Thank you for the opportunity to review this manuscript. It was very well written, easy to follow, and is an important contribution to the literature. I have suggested only minor revisions as detailed below. | Thank you for taking the time to review our manuscript. We greatly appreciate it as we know reviews can be time consuming! |
| Introduction – Very well written; clear and easy to follow. The introduction develops the important narrative necessary for background understanding of the present study. | Thank you again. |
| Methods – Would the authors please report the number of individuals that were contacted by Ipsos to complete the survey so that the reader can gauge the response rate? If that information is not available, please say so. | Great question. The response rate was not available but a comment stating such was added along with commentary on steps to address non-response bias and a citation for the methodological report if readers care to read further. |
| The authors should also provide details on the length of the survey (e.g., was it just the questions for this study or were there other items included for other studies?). | Thank you for this comment. Multiple sets of questions were asked covering a range of topics unknown to our research group. A comment clarifying this was added to the Survey Outcomes section. |
| Further, would the authors please report whether there were key demographic factors that were associated with missing data on the received surveys? For example, were older individuals less likely to complete the instrument? | Great question. Sensitivity analyses revealed no systematic differences between the included data and missing data. Revised the comment about the sensitivity analyses on all variables at the end of the Results section. |
| Why were the worry-based items graded on a 4-point Likert scale when the belief-based items were rated on 5-point Likert scales? | This is a good question. The parental worry questions were a separate set of items, all of which were measured on a four-point Likert scale with these response options: I don’t worry at all, I worry only a little, I worry some, I worry a lot. Other questions were asked as a set of agree/disagree statements that had these response options: Strongly disagree, Disagree, Neither agree nor disagree, Agree, Strongly Agree. In our next study on this topic we will be revisiting the item structure based on this exploratory data. We added a clarifying comment to the end of the “worry about head injuries” methodological section. |
| Would the authors please clarify if the education level of “attended college (62.5%)” refers only to those who completed a degree program, or if it includes those who may have attended some college without obtaining a post-secondary degree? | Thanks for clarifying. We added “(i.e., with or without obtaining a degree).” |
| Why was household income dichotomized at $75k? Was this a median split? | Good question, that’s exactly right, a median split. Added the original/raw demographic categories to an Appendix to increase transparency and reproducibility. |
| Results – For transparency’s sake, I would encourage the authors to also report the frequency of each Likert scale response to all questions, either in Table 1 or in supplemental materials. I understand this is asking for some tedious work, but feel it may be useful for yours and others’ future work that may utilize similar questionnaires. I apologize if this has already been done; I was unable to successfully open the link to the appendix in the review pdf file. | Excellent suggestion. We have created and uploaded this information with frequencies and percentages in a new Supplemental Table 1. |
| I’m assuming the beta values in Table 2 are standardized. Would you please make this clear in the table note and in the main text where “Est = “ is presented? | Thank you for pointing this out. Adjustment made in Table 2 note. We believe this will add clarity but if additional clarification is desired we will be happy to add it elsewhere. |
| Discussion – The authors note that one possible explanation for overestimated concussion risk is that it might be associated with increased media attention. Were the associations between independent variables evaluated? Specifically, does perception of greater media exaggeration associate with lower perceived concussion risk in your sample? On a related note, did the authors consider interaction effects among independent variables (e.g., parentage and worries, for example)? | Correlations between all variables were evaluated. There was not a strong correlation between perception of greater media exaggeration and perceived concussion risk (correlation was -.23). Great question about interactions. We chose to not explore interactions because we did not have any that were of specific interest to our hypotheses. We did not want to probe variables that we did not plan for a priori. |
| Thank you for addressing the concern regarding only asking about boys’ participation in football in your note. | Thank you for this comment. We added this important note content into the manuscript so that the superscript/note would not be missed by readers. |
