# Supplementary material for: Factors associated with U.S. adults’ willingness to allow teenagers to play tackle football
Source: PLoS One. 2022 Sep 7;17(9):e0273229. doi: 10.1371/journal.pone.0273229 (PMC9451093; doi:10.1371/journal.pone.0273229)
Supplement: Attachment — Submitted filename: Response to reviewer comments.docx [file pone.0273229.s008.docx]

| **Response to Reviewers** | |
| --- | --- |
| **Reviewer Comment** | **Response / Revision** |
| Reviewer #1: Thank you for the thorough revision and addressing all initial points. This reviewer feels the manuscript is acceptable as is (pending copyediting) for publication. The only additional consideration is PLOS One asks that data be made publicly available. The authors indicated "yes", but their comment on this is that "All relevant data are within the manuscript and its Supporting Information files." However, PLOS Ones policy on this is that this does not constitute data being publicly available (i.e. they want the underlying, individual datapoints/dataset be provided). If this is not possible due to IRB or other concerns, the authors should revise their statement to avoid any confusion. | Thank you for these comments and for your thoughtful reviews during this process. They were greatly appreciated. To address your concern, we have added the entire raw dataset to the uploaded files. |
| Reviewer #2: Thank you for addressing my comments and those of the other reviewer. I have no further requests for revision at this time. | Thank you again for reviewing this manuscript. |
| Editor comment: After careful consideration of your research question, results, and discussion further revision is needed. More specifically, the malalignment between the manuscript narrative, primary research question, methodology and subsequent results and discussion should be focused solely on tackle football opposed to physical activity in general prior to consideration for publication. As your survey instrument is focused solely on tackle football, the manuscript should be adjusted to focus solely on this sport opposed to "sport" or "physical activity" in general. In sum, your important findings are based solely related to tackle football opposed to sport in general and the narrative should be revised to focus on this theme. | Thank you for these thoughtful suggestions. We are happy to adjust the manuscript narrative as requested. We have either deleted commentary on *physical activity* and *sports* in general or edited the text to refer to *tackle football/football* instead of *sport/sports*.  These adjustments are summarized below (see Revised Manuscript with Track Changes for matching line numbers). There were a few instances where we kept the word sport or sports where we thought it made logical sense or were referring to a specific study that was referencing many sports, but overall, we believe we have fixed the malalignment. Please let us know if you feel otherwise and we would be happy to make additional edits. Thank you kindly!  For the **Introduction**, which is where much of the narrative was about sport or physical activity, we deleted almost the entire the first paragraph and much of the second paragraph. The narrative specifically about *physical activity* has been removed. Out of this 1.5 paragraph deletion, we kept 3 sentences that we felt helped introduce the importance of the manuscript (e.g., nationally representative sample) without overstepping to physical activity recommendations.  We also introduced tackle football immediately (in the first paragraph) instead of discussing sports and physical activity more broadly. If you would like these 3 initial sentences removed please let us know.  **Page 2, Abstract, lines 18-21:** First 2 sentences about *sports* and *physical activity* deleted. **Line 23:** *sports* changed to *tackle football*. **Line 33:** deleted *sport*. **Line 35-36:** changed *youth sport and physical activity* to *tackle football*.  **Page 4, line 70-71:** changed *youth sport participation* to *in youth sports, such as tackle football*.  **Page 16, line 294-296:** Deleted Discussion intro sentence broadly about sport and physical activity.  **Page 17, line 299:** changed *sports* to *tackle football*. **Line 303-304:** kept the term “youth sport participatory research” since the referenced studies were addressing *sports* rather than just *tackle football.* **Line 314**: *sport policy* changed to *football safety policy.*  **Page 18, line 329:** *sport and/or physical activity* changed to *football*. **Line 331:** *sport participation* changed to *football participation*.  **Page 19, line 358:** changed *youth sport participation* to *youth football participation*.  **Page 22, line 409:** *youth sport participatory outcomes* changed to *youth football participatory outcomes*. **Line 409-410:** *youth sport research* changed to *youth football research*.  **Page 22, line 414:** deleted *sport*. **Line 417:** changed *sports* to *football*. |
